# Common Phenolic Metabolites of Flavonoids, but Not Their Unmetabolized Precursors, Reduce the Secretion of Vascular Cellular Adhesion Molecules by Human Endothelial Cells^1^,^2^,^3^

**DOI:** 10.3945/jn.115.217943

**Published:** 2016-02-03

**Authors:** Emily F Warner, Qingzhi Zhang, K Saki Raheem, David O’Hagan, Maria A O’Connell, Colin D Kay

**Affiliations:** 4 Department of Nutrition, Norwich Medical School, and; 5 School of Pharmacy, University of East Anglia, Norwich Research Park, Norwich, United Kingdom;; 6 School of Chemistry, University of St. Andrews, St. Andrews, United Kingdom; and; 7 Department of Life Sciences, Faculty of Science and Technology, University of Westminster, London, United Kingdom

**Keywords:** polyphenol, VCAM-1, metabolism, endothelial, inflammation, phase II conjugate

## Abstract

**Background:** Flavonoids have been implicated in the prevention of cardiovascular disease; however, their mechanisms of action have yet to be elucidated, possibly because most previous in vitro studies have used supraphysiological concentrations of unmetabolized flavonoids, overlooking their more bioavailable phenolic metabolites.

**Objective:** We aimed to explore the effects of phenolic metabolites and their precursor flavonoids at physiologically achievable concentrations, in isolation and combination, on soluble vascular cellular adhesion molecule-1 (sVCAM-1).

**Method:** Fourteen phenolic acid metabolites and 6 flavonoids were screened at 1 μM for their relative effects on sVCAM-1 secretion by human umbilical vein endothelial cells stimulated with tumor necrosis factor alpha (TNF-α). The active metabolites were further studied for their response at different concentrations (0.01 μM–100 μM), structure-activity relationships, and effect on vascular cellular adhesion molecule (*VCAM*)*-1* mRNA expression. In addition, the additive activity of the metabolites and flavonoids was investigated by screening 25 unique mixtures at cumulative equimolar concentrations of 1 μM.

**Results:** Of the 20 compounds screened at 1 μM, inhibition of sVCAM-1 secretion was elicited by 4 phenolic metabolites, of which protocatechuic acid (PCA) was the most active (−17.2%, *P* = 0.05). Investigations into their responses at different concentrations showed that PCA significantly reduced sVCAM-1 15.2–36.5% between 1 and 100 μM, protocatechuic acid-3-sulfate and isovanillic acid reduced sVCAM-1 levels 12.2–54.7% between 10 and 100 μM, and protocatechuic acid-4-sulfate and isovanillic acid-3-glucuronide reduced sVCAM-1 secretion 27.6% and 42.8%, respectively, only at 100 μM. PCA demonstrated the strongest protein response and was therefore explored for its effect on *VCAM-1* mRNA, where 78.4% inhibition was observed only after treatment with 100 μM PCA. Mixtures of the metabolites showed no activity toward sVCAM-1, suggesting no additive activity at 1 μM.

**Conclusions:** The present findings suggest that metabolism of flavonoids increases their vascular efficacy, resulting in a diversity of structures of varying bioactivity in human endothelial cells.

## Introduction

Epidemiological studies have demonstrated associations between diets high in flavonoid-rich foods and the reduced risk of cardiovascular disease (1–3). Furthermore, the protective effects of dietary flavonoids have been observed in numerous randomized control trials (4–8) and animal feeding studies (9–14). Unfortunately, the direct mechanisms of action of flavonoids have been elusive. Much of the focus of previous studies has been on direct vascular reactivity, affecting blood pressure, blood flow, heart rate variability, and flow-mediated vasodilation (15, 16); however, low-level chronic inflammation, attributed to the expression of vascular adhesion molecules on the surface of the endothelium, has long been implicated as a driving factor in the early stages of atherosclerosis (17, 18). TNF-α is a cytokine that serves as a mediator in a number of diseases, such as atherosclerosis, and stimulates the production of a number of pro-inflammatory biomarkers (19), such as circulating levels of soluble vascular adhesion molecule-1 (sVCAM-1)^8^, an important predictor of risk of death from coronary heart disease (20). TNF-α–stimulated sVCAM-1 expression therefore provided a logical target for exploring the potential mechanisms of action of flavonoid metabolites in the present investigation. The present study used human umbilical vein endothelial cells (HUVECs), which are an established model for the study of endothelial dysfunction, with similar expression profiles to arterial endothelial cells in response to inflammatory stimuli (21).

**FIGURE 1:**
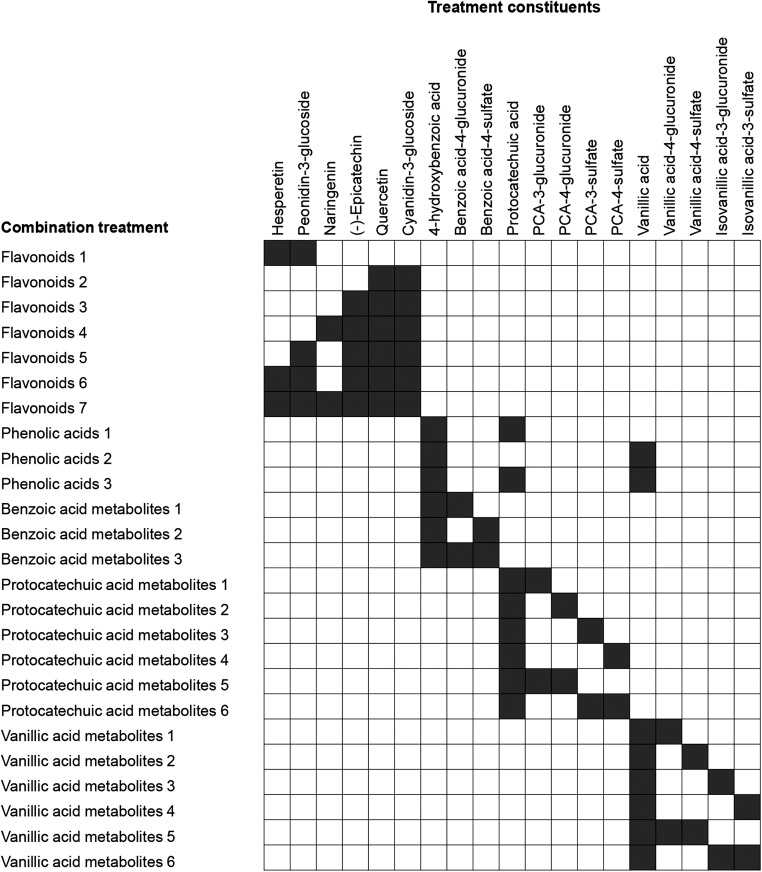
Combination treatments used in this study. Shaded boxes represent inclusion of respective compounds in equimolar concentrations to a cumulative concentration of 1 μM; for example, a combination comprising 4 constituents would require 0.25 μM of each to yield a final concentration of 1 μM. PCA, protocatechuic acid.

Previous studies have demonstrated potentially beneficial effects of flavonoids on some inflammatory mechanisms in vitro, including inhibition of the adhesion of leukocytes to endothelial cells (22–24). Unfortunately again, the mechanisms underlining these effects are unknown, potentially because previous in vitro investigations have focused on the activity of unmetabolized flavonoids, which are found in relatively low abundance in the circulation compared with their metabolites, and have considerably shorter half-lives (25–28). It has therefore been suggested that the biological activity observed in human studies results from the activity of products of bacterial catabolism, absorption, and further phase II metabolism (29, 30), which were the focus of the present study. In addition, many past in vitro studies have used supraphysiological concentrations of precursor/unmetabolized flavonoids, while only a limited few have reported the activity of free-phenolic acids (31). Until recently (32, 33) few have explored the activity of phase II conjugates of phenolic acid derivatives (34–37), primarily as a result of the lack of availability of synthetic standards (29, 38). We hypothesized that phenolic metabolites of flavonoids will have differential biological activities to their precursor structures and that metabolites in combination may have additive or synergistic effects. We therefore screened 6 flavonoids found commonly in the Western diet (**Supplemental Figure 1**); 14 human metabolites, as previously reported (25–27, 39); and 25 combinations of the flavonoids and their metabolites (at equimolar concentrations) (**Figure 1**), for their ability to reduce sVCAM-1 protein secretion by TNF-α–stimulated HUVECs. Investigations into the response to different concentrations of active treatments were also explored, including 4 physiological (between 0.01 μM and 10 μM) and 1 supraphysiological (100 μM) concentration. The most active treatment was further assessed for its activity on transcription regulation via mRNA expression of vascular cellular adhesion molecule (*VCAM*)*-1*.

## Methods

### 

#### Materials.

Early passage HUVECs (cryopreserved, pooled donors), large vessel endothelial growth medium (containing 2% fetal calf serum, human epidermal growth factor, human fibroblast growth factor, 25 μg/mL gentamycin, 50 μg/L amphotericin, hydrocortisone, and heparin), and trypsin passage pack were purchased from Caltag Medsystems (Buckingham, United Kingdom). Human-derived fibronectin, TNF-α, and BAY 11–7085 were purchased from Sigma Aldrich (Dorset, United Kingdom). The conjugated metabolites, as listed in Supplemental Figure 1, were synthesized at the University of St. Andrews (United Kingdom) (40). All flavonoids and unconjugated phenolic acids were obtained from Sigma Aldrich (Dorset, United Kingdom), with the exception of peonidin-3-glucoside (Extrasynthase, France).

#### Preparation of treatment solutions.

Stock solutions of all compounds screened were prepared in 100% DMSO at 200 mM and stored at −80°C with the exception of cyanidin-3-glucoside and peonidin-3-glucoside, which were prepared at 40 mM, and the sulfate-conjugated phenolic acids, which were prepared at 25 mM in 50% DMSO (50% PBS) to maintain stability while reducing final DMSO concentrations in working solutions. Working solutions of all treatments were made in supplemented media. Treatments containing mixtures of compounds (Figure 1) consisted of equimolar concentrations of the constituent treatment compounds to a cumulative concentration of 1 μM.

#### Cell culture.

HUVECs were maintained in supplemented large vessel endothelial medium on fibronectin-coated cell culture plates (0.25 μg/cm^2^) at 37°C, 5% CO_2_, in a humidified atmosphere, as previously described (40). Cells were reseeded at 90–95% confluence using a trypsin passage pack. HUVECs were used at passage 4 in all experiments.

#### Cell treatment and stimulation.

HUVECs were seeded into fibronectin-coated 24-well cell culture plates (0.25 μg/cm^2^) and incubated in supplemented media for 24 h before treatment. Media was then removed and replaced by 1 μM treatment solutions for the screen of individual compounds and treatment mixtures (1 μM cumulatively) and 0.01–100 μM for protein and mRNA concentration response experiments. IκBα inhibitor BAY 11–7085 (41) was included as a negative control for all mRNA experiments. Cells were incubated with treatment solutions for 30 min before the addition of TNF-α (10 μg/L) for 18 h, as used in previous studies (42, 43). A vehicle control of equivalent DMSO concentration was used in each experiment, where the concentration did not exceed 0.1%, as was the control for 100 μM solutions, and did not exceed 0.02% for all other treatments. Cell culture supernatants were collected and used immediately or stored at −80°C until required.

#### sVCAM-1 ELISA.

Human sVCAM-1 protein levels in recovered cell culture supernatants were assayed using a Human VCAM-1/CD106 DuoSet ELISA kit (R&D Systems; Abingdon, United Kingdom), according to the manufacturer’s instructions. sVCAM-1 was quantified via colorimetric assay at 450 nm, corrected for 570 nm, using a BMG (LABTECH) plate reader. The interassay coefficient of variation was 8.8%.

#### RNA extraction, reverse transcription, and real-time qPCR.

Total RNA was extracted from HUVECs and reverse transcribed to cDNA using conditions previously described by this group (32). Real-time qPCR was carried out using 25 ng of cDNA of each sample, with the addition of *VCAM-1* primers (forward primer, 5′-CAGGCTAAGTTACATATTGATGACAT-3′; reverse primer, 5′-GAGGAAGGGCTGACCAAGAC-3′) and real time PCR Precision master mix with SYBR green (Primer Design). Real-time qPCR was carried out using the ABI7500 system, using cycle methods previously described (32). Relative changes in gene expression from the TNF-α control were quantified using the comparative Ct method (44). The difference between recorded Ct values for treatment and positive control was calculated in the first instance for all genes. *VCAM-1* values were normalized to 2 geNORM housekeeping genes, *UBE2D2* and *PRDM4* (Primer Design), selected based on their stability, as established using qPCR data analysis software qbase^PLUS2^ (Biogazelle, Belgium), where the geometric mean of the 2 housekeeping genes was used as the normalization factor (45).

#### Data analysis.

sVCAM-1 protein (in pg/mL) or mRNA (fold change) were recorded as the mean of 2 technical duplicates and reported relative to the TNF-α positive control (containing TNF-α without DMSO), where data represents the mean ± SD of 3 independent measures (*n* = 3). Unequal variances were tested by use of Levene’s test, where the null hypothesis was rejected at the level of 0.05. Treatments containing combinations of metabolites were identified as nonparametric, whereas all other treatments satisfied Levene’s criteria. Where unequal variances were identified (i.e., treatments containing combinations of metabolites), between-group differences were established via Kruskal-Wallis ANOVA. Treatment effects for parametric variables were established by 1-factor ANOVA with post hoc least square difference. Analysis was conducted using SPSS for Windows (version 22.0; IBM). Data were considered significant where *P* ≤ 0.05. Untreated and negative controls were not included in the ANOVA for treatment effect but presented graphically, where a Student’s *t* test established difference relative to the vehicle control (DMSO). For screening purposes, treatments displaying nonsignificant values ≤0.15 were taken forward, for validation in subsequent concentration analysis.

## Results

### 

#### Effects of flavonoid and flavonoid metabolites on sVCAM-1 secretion.

**FIGURE 2:**
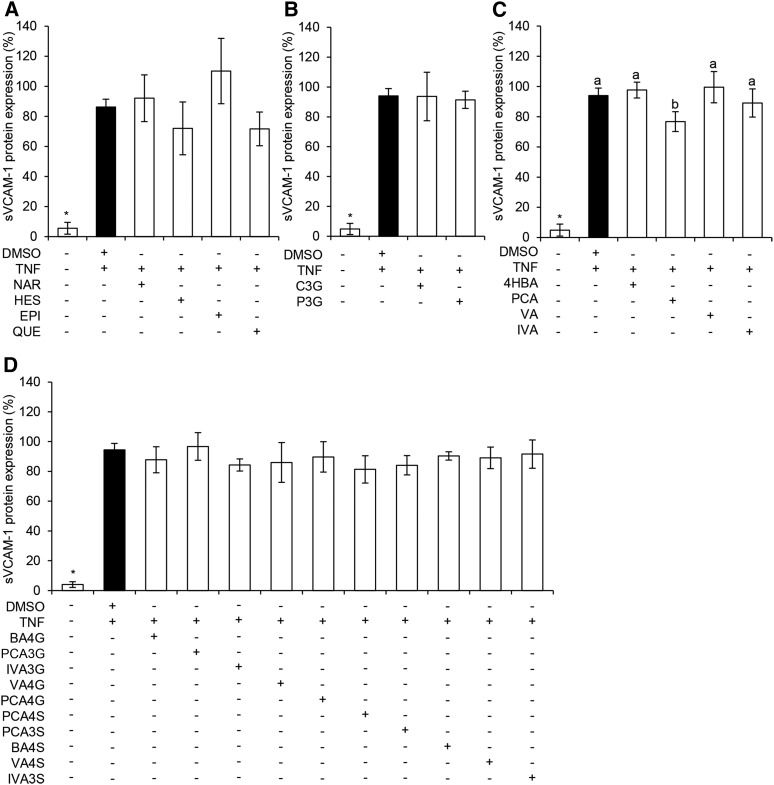
Effect of 1 μM flavonoids and phenolic acid metabolites on TNF-α–stimulated sVCAM-1 protein secretion by HUVECs. Flavonoids (A), anthocyanin glucosides (B), unconjugated phenolic acids (C), and conjugated phenolic acids (D) are shown. Data were normalized to a TNF-α control, and columns represent the mean ± SD (*n* = 3 independent measures). Labeled means without a common letter differ, *P* ≤ 0.05 (ANOVA with post hoc LSD). *Different from DMSO, *P* ≤ 0.05 (*t* test). BA4G, benzoic acid-4-glucuronide; BA4S, benzoic acid-4-sulfate; C3G, cyanidin-3-glucoside; EPI, (-) epicatechin; HES, hesperetin; HUVEC, human umbilical vein endothelial cell; IVA, isovanillic acid; IVA3G, IVA-3-glucuronide; IVA3S, IVA-3-sulfate; LSD, least square difference; NAR, naringenin; PCA, protocatechuic acid; PCA3G, PCA-3-glucuronide; PCA3S, PCA-3-sulfate; PCA4G, PCA-4-glucuronide; PCA4S, PCA-4-sulfate; P3G, peonidin-3-glucoside; QUE, quercetin; sVCAM-1, soluble vascular cellular adhesion molecule 1; VA, vanillic acid; VA4G, VA-4-glucuronide; VA4S, VA-4-sulfate; 4HBA, 4-hydroxybenzoic acid.

**FIGURE 3:**
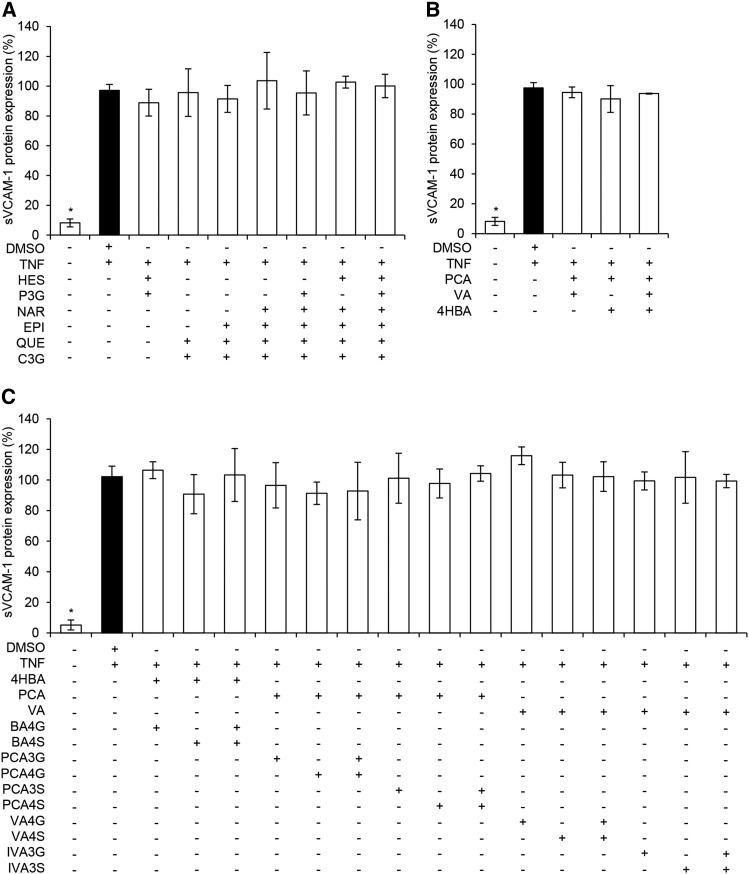
Effect of 1 μM mixtures of flavonoids and phenolic acid metabolites on TNF-α stimulated sVCAM-1 protein secretion by HUVECs. Flavonoid mixtures (A), phenolic acid mixtures (B), and conjugated and unconjugated phenolic metabolite mixtures (C) are shown. Data were normalized to a TNF-α control, and columns represent the mean ± SD (*n* = 3 independent measures). *Different from DMSO, *P* ≤ 0.05 (*t* test). Where unequal variance was identified (B and C), group differences were established via Kruskal-Wallis nonparametric ANOVA. BA4G, benzoic acid-4-glucuronide; BA4S, benzoic acid-4-sulfate; C3G, cyanidin-3-glucoside; EPI, (-) epicatechin; HES, hesperetin; HUVEC, human umbilical vein endothelial cell; IVA3G, isovanillic acid-3-glucuronide; IVA3S, isovanillic acid-3-sulfate; NAR, naringenin; PCA, protocatechuic acid; PCA3G, PCA-3-glucuronide; PCA3S, PCA-3-sulfate; PCA4G, PCA-4-glucuronide; PCA4S, PCA-4-sulfate; P3G, peonidin-3-glucoside; QUE, quercetin; sVCAM-1, soluble vascular cellular adhesion molecule 1; VA, vanillic acid; VA4G, VA-4-glucuronide; VA4S, VA-4-sulfate; 4HBA, 4-hydroxybenzoic acid.

Six flavonoids and 14 phenolic metabolites were screened at a concentration of 1 μM for their ability to reduce TNF-α–stimulated sVCAM-1 secretion by HUVECs. Precursor flavonoids had no significant effect on sVCAM-1 secretion, although there was a moderate, nonsignificant increase in the secretion of sVCAM-1 (*P* = 0.14) following treatment with (-)-epicatechin (**Figure 2**). The metabolite protocatechuic acid (PCA) significantly decreased sVCAM-1 secretion (*P* = 0.05), whereas nonsignificant effects were observed for treatments with sulfate [protocatechuic acid-4-sulfate (PCA4S), *P* = 0.07; protocatechuic acid-3-sulfate (PCA3S), *P* = 0.14] and glucuronide [isovanillic acid-3-glucuronide (IVA3G), *P* = 0.15] conjugates of PCA. Treatments showing the greatest activity (*P* ≤ 0.15) were taken forward to explore response at different concentrations. Seven treatments containing mixtures of flavonoids and 18 treatments containing mixtures of phenolic metabolites were also investigated for their effect on sVCAM-1 secretion (**Figure 3**); however, no activity was observed at a cumulative concentration of 1 μM of the compounds (*P* ≥ 0.27), and therefore no combination treatments were taken forward for analysis of response to different concentrations.

#### Response to different concentrations of active metabolites on sVCAM-1 secretion.

**FIGURE 4:**
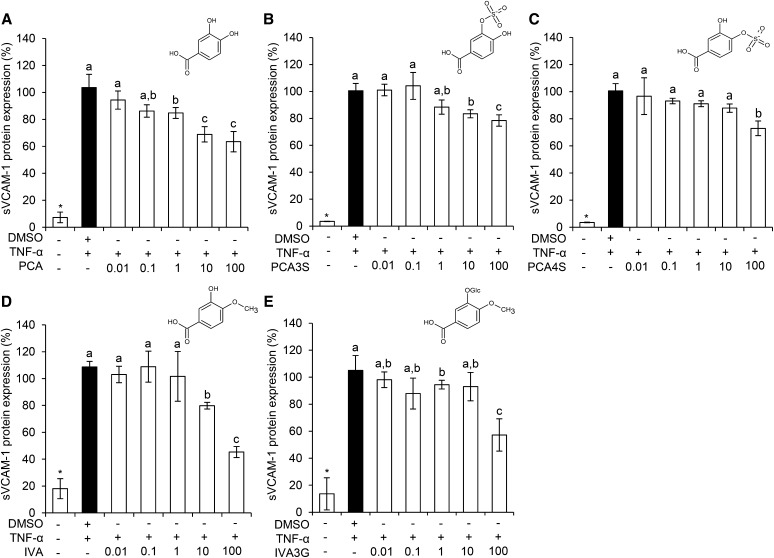
Effect of concentration of phenolic acid metabolites on TNF-α–stimulated sVCAM-1 protein secretion by HUVECs. PCA (A), PCA3S (B), PCA4S (C), IVA (D), and IVA3G (E) are shown. Data were normalized to a TNF-α control, and columns represent the mean ± SD (*n* = 3 independent measures). Labeled means without a common letter differ, *P* ≤ 0.05 (ANOVA with post hoc LSD). *Different from DMSO, *P* ≤ 0.05 (*t* test). HUVEC, human umbilical vein endothelial cell; IVA, isovanillic acid; IVA3G, IVA-3-glucuronide; LSD, least square difference; PCA, protocatechuic acid; PCA3S, PCA-3-sulfate; PCA4S, PCA-4-sulfate; sVCAM-1, soluble vascular cellular adhesion molecule 1.

sVCAM-1 secretion was investigated following treatment with 0.01 μM–100 μM of the most active treatments PCA, PCA3S, PCA4S, and IVA3G (**Figure 4**). Isovanillic acid (IVA), although not active in the sVCAM-1 protein screen, was also included in order to establish structure-activity relationships (SARs) with PCA and IVA conjugates. Here, PCA significantly reduced sVCAM-1 levels in a concentration-dependent manner at concentrations between 1 μM and 100 μM, whereas PCA3S and IVA were active at levels between 10 μM and 100 μM, and PCA4S and IVA3G were only active at 100 μM.

#### Response to different concentrations of PCA on *VCAM-1* mRNA expression.

**FIGURE 5:**
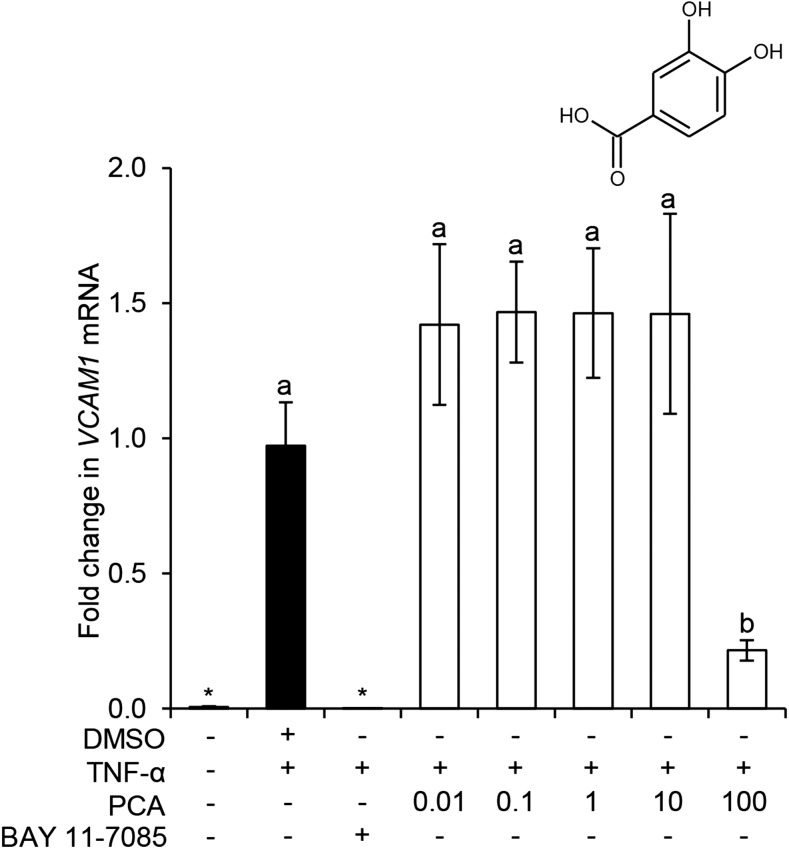
Effect of concentration of PCA on TNF-α–stimulated *VCAM-1* mRNA expression in HUVECs. Data were normalized to a TNF-α control, and columns represent the mean ± SD (*n* = 3 independent measures). Labeled means without a common letter differ, *P* ≤ 0.05 (ANOVA with post hoc LSD). *Different from DMSO, *P* ≤ 0.05 (*t* test). HUVEC, human umbilical vein endothelial cell; LSD, least square difference; PCA, protocatechuic acid; VCAM, vascular cellular adhesion molecule.

Because PCA showed the highest activity on protein secretion, and this effect was amplified with increased concentration, we further investigated whether this response was reciprocated in *VCAM-1* mRNA expression (**Figure 5**). Here, TNF-α significantly induced *VCAM-1* mRNA expression after 4 h (*P* ≤ 0.01), and this effect was fully inhibited by treatment with the negative control (IκBα-inhibitor BAY 11–7085). Treatment with 100 μM PCA was the only concentration to significantly inhibit *VCAM-1* mRNA expression (78% inhibition; *P* = 0.05).

## Discussion

The consumption of flavonoid-rich foods has been associated with reduced cardiovascular disease risk (1–3), yet their mechanisms of action have yet to be elucidated. It has been suggested that the beneficial effects of flavonoids are the result of degradation products (chemical degradation or bacterial catabolites) and phase II metabolites (29, 30). We investigated phenolic metabolites commonly reported for berry anthocyanins (39, 46), cocoa and tea (47–50), and citrus fruits (51), focusing on metabolites that differed primarily on their 3′ and 4′ positions (relative to the precursor B-ring structure) to draw conclusions regarding SARs. The focus on physiologically achievable concentrations of metabolites in cell culture studies is reasonably contemporary (29, 52) , whereas investigation of the additive activities of conjugated and unconjugated metabolites in combination, at concentrations achievable through diet, is novel. We investigated the differential effects of 20 flavonoids and common metabolites and 25 combinations thereof. We found that 4 phenolic metabolites modulated sVCAM-1 secretion, whereas no effects were observed for their precursor structures. The response to PCA appeared amplified with increasing concentration, which is suggestive of a dose-dependent response. Inhibition of *VCAM-1* mRNA was observed in response to PCA; however, this was only at a supraphysiological concentration of 100 μM. Furthermore, mixtures of metabolites and flavonoids showed no activity toward sVCAM-1, suggesting no additive activity.

sVCAM-1 was a logical target to investigate the vasoprotective activity of flavonoids, because it is a clinical predictor of risk of death from cardiovascular disease (20) and previous studies have demonstrated beneficial effects of flavonoids on adhesion of leukocytes to endothelial cells (22–24). These findings are supported by additional recent studies by our group, where metabolites were active on IL-6 and sVCAM-1 production following stimulation with CD40 and oxidized LDL in vascular endothelial cells and TNF-α following LPS stimulation in human monocytes (32, 33).

Five treatments (Figure 4) were further explored for the effect of increased concentration on sVCAM-1 protein; here, 1 metabolite (PCA) significantly inhibited sVCAM-1; 3 (PCA3S, PCA4S, IVA3G) had moderate, nonsignificant activity; and 1 (IVA) was selected to draw conclusions regarding SARs between PCA and IVA conjugates. Of the compounds screened, PCA was most active across the concentrations tested, which is in line with previous studies, where it was observed to inhibit the expression of inflammatory mediators, including adhesion molecules (53, 54). The activity of PCA was comparable to its aldehyde equivalent (protocatechuic aldehyde), in a study identifying a concentration-dependent reduction in TNF-α–stimulated sVCAM-1 (42); this supports the premise that the catechol moiety of flavonoid metabolites holds significant activity (55). However, given PCAs reactive catechol moiety is rapidly methylated by catechol-O-methyltransferase (56), it does not persist in the systemic circulation at any appreciable concentration for significant periods of time (25, 39, 46), whereas its metabolite, vanillic acid, exists at much higher concentrations and has a considerably longer half-life (25, 39, 46, 51). Vanillic acid may therefore make an appropriate target for future investigation, given that it was recently shown to have a significant effect on CD40-stimulated *VCAM-1* mRNA (32).

The lack of dietary relevance of contemporary cell culture studies in the field of nutrition is apparent, given the use of precursor structures at supraphysiological concentrations, which may explain why the underlying mechanisms of action are still unknown (29). It is interesting that we observed significant inhibition of sVCAM-1 in response to PCA at 1 μM, because previous studies have identified serum concentrations of PCA ranging between 0.15 μM (25) and 1.5 μM (46, 57), suggesting this effect is achievable through diet.

Given the apparent strength and linearity of the concentration responsiveness of PCA, we sought to explore if this was reflected in the expression of *VCAM-1* mRNA (Figure 5), as advocated by others (32, 58). Here, PCA was only active at 100 μM, suggesting PCA is not directly active on mRNA transcription at physiologically achievable concentrations, but likely acting post-translationally. It is conceivable metabolites could interact with the cleavage of the protein from the surface of endothelial cells (59), such as by interaction with TNF-α converting enzyme ADAM17 (60), an indicated mediator of VCAM-1 shedding from the surface of endothelial cells. Future studies exploring the mechanisms of action of PCA should therefore focus on post-translational or receptor-binding activities.

Investigations of SARs are important for understanding how metabolism alters phytochemical activity. Because previous studies have reported the SAR of flavonoids (61, 63), we aimed to draw conclusions based on relationships between conjugated and unconjugated phenolic metabolites (**Supplemental Figure 2**). Of the 5 metabolites studied, PCA had the greatest effect on sVCAM-1, with PCA3S, PCA4S, and IVA having equally lesser activity and IVA3G having no effect, suggesting conjugation of both the hydroxyl moieties reduces potency on sVCAM-1 secretion. Conjugation has also been shown to reduce the inhibitory activity of certain flavonoids on monocyte adhesion (52, 55); however, the opposite has recently been reported in oxidized-LDL–stimulated HUVECs, where conjugation of PCA increased the inhibition of sVCAM-1 (32), suggesting the effects of conjugation are dependent on the inflammatory stimulus, and thus the upstream signal transduction pathway involved, as suggested for other flavonoids (63).

After ingestion, flavonoids circulate as complex mixtures of metabolites at various concentrations (26, 39, 64), thus it is important that this is reflected in cell culture experiments. Few studies have explored the effects of flavonoids in combination, despite indication of differential activities when in combination relative to isolation (65, 66). The present study examined 25 mixtures of equimolar concentrations of structurally similar compounds. Treatments were designed in this manner because plasma concentrations of flavonoid/metabolites vary greatly between, and indeed within, subjects. Therefore, it was deemed unrealistic to model treatments mimicking human plasma compositions identified in any single feeding study. Here we did not observe any inhibitory effects on sVCAM-1 secretion from mixtures totaling 1 μM in concentration (cumulative concentration of analytes present in an equimolar ratio). This represented concentrations of each analyte between 0.5 and 0.17 μM, and although these concentrations represent physiologically relevant levels, it is possible that they were too low to elicit a quantifiable response. A recent human study identified total phenolic metabolites as high as 13.3 μM following consumption of orange juice flavanones (51), suggesting greater cumulative concentrations are indicated in future cell culture studies. Given that mixtures of phenolic metabolites have shown differential effects to their constituents in isolation in LPS stimulated THP-1 cells (33), cumulative effects of these metabolites may be cell-type specific.

Here we provide insight into the differential activity of flavonoid metabolites and have explored their potential for additive effects; however, there are certain limitations of this work. The use of soluble VCAM-1 over membrane-bound VCAM-1 could be a limitation because it is the membrane-bound VCAM-1 that binds directly to leukocytes in the progression of atherosclerosis (18). However, it has been reported that sVCAM-1 protein levels directly correlate with levels of surface-bound VCAM-1 (67) and may be a more appropriate biomarker of endothelial cell activation (59); that said, further investigation of the relative activity of these metabolites on surface-bound VCAM-1 would verify such correlations (67). The concentration of TNF-α used to stimulate sVCAM-1 could also be viewed as a limitation. The concentration 10 μg/L was selected because it is commonly reported in the literature (41, 42, 68, 69); however, physiologically, plasma concentrations are reported at 0.001–0.04 μg/L in patients with coronary artery disease (70) and can reach 2 μg/L in patients who have suffered myocardial infarction (71). Future studies could therefore consider using models that more closely reflect in vivo conditions. The stimulation time used in our pro-inflammatory model could also be considered a limitation. As previously discussed, the design of the treatment mixtures used in this investigation was quite artificial, because equimolar ratios would not mimic serum concentrations observed in human studies; future studies could therefore use metabolites at a range of concentrations, based on levels reported in human feeding studies (25–27). Finally, HUVECs do not originate from the arterial walls, and therefore their use may be considered a limitation. Verification was carried out using single donor, human coronary artery endothelial cells, where induction of VCAM-1 in response to TNF-α was greater than in HUVECs; however, the response to increasing concentrations of PCA was similar in both cell types (data not shown).

The present study supports previous reports that metabolism of flavonoids to phenolic acids alters their anti-inflammatory effects (61, 72–74). Our data indicate that the degradation of flavonoids to phenolic acids, which is believed to be largely facilitated by microbiota in the colon (25, 64), increases their overall bioactivity, whereas further conjugation by phase II enzymes may have differential effects on activity (32, 33). Therefore, certain flavonoids consumed in our habitual diet may require prior metabolism before they can exert their maximal effects and metabolites may possess differential bioactivities as they are systematically metabolized and eliminated from the circulation.

In conclusion, the present study provides insight into the activity of conjugated and unconjugated phenolic metabolites of flavonoids, thus contributing to our understanding of how these dietary phytochemicals influence cardiovascular health.

## Footnotes

8 Abbreviations used: HUVEC, human umbilical vein endothelial cell; IVA, isovanillic acid; IVA3G, isovanillic acid-3-glucuronide; PCA, protocatechuic acid; PCA3S, protocatechuic acid-3-sulfate; PCA4S, protocatechuic acid-4-sulfate; SAR, structure-activity relationship; sVCAM-1, soluble vascular cellular adhesion molecule 1; VCAM, vascular cellular adhesion molecule.
